# “In Flow”! Why Do Users Share Fake News about Environmentally Friendly Brands on Social Media?

**DOI:** 10.3390/ijerph19084861

**Published:** 2022-04-16

**Authors:** Daniel-Rareș Obadă, Dan-Cristian Dabija

**Affiliations:** 1 Department of Communication Sciences and Public Relations, Faculty of Philosophy and Socio-Political Sciences, Alexandru Ioan Cuza University of Iași, 700506 Iași, Romania; 2 Department of Marketing, Faculty of Economics and Business Administration, Babeș-Bolyai University, 400591 Cluj-Napoca, Romania; dan.dabija@ubbcluj.ro

**Keywords:** fake news, social media platforms, social media flow, social media usage, flow theory, environmentally friendly brands, user behavior, online sharing news, online trust, structural equations modeling

## Abstract

Social media has triggered an increase in fake news spread about different aspects of modern lives, society, politics, societal changes, etc., and has also affected companies’ reputation and brands’ trust. Therefore, this paper is aimed at investigating why social media users share fake news about environmentally friendly brands. To examine social media users’ behavior towards environmentally friendly brands, a theoretical research model proposed and analyzed using structural equations modeling in SmartPLS on a convenience sample consisting of 922 questionnaires. Data was collected by means of a quantitative-based approach via a survey conducted among social media users from an emerging market. The results show that social media flow has a mediated impact on sharing fake news about environmentally friendly brands on social media. Considering the critical consequences of fake news, the paper argues that understanding the dissemination process of this type of bogus content on social media platforms has important theoretical and managerial implications. Understanding the psychological mechanisms that influence people’s behavior in sharing fake news about environmentally friendly brands on social networking sites (SNS) could help in better understanding the factors and the effects of this phenomenon. The originality of this research consists of proposing flow theory from positive psychology to be used as a theoretical framework to explain users’ behavior of sharing fake news about environmentally friendly brands on social media.

## 1. Introduction

The number of internet users has expanded significantly in recent years, reaching 4.95 billion in January 2022, representing a 65.6% penetration rate in the world population. The global number of active social media users was estimated to be roughly 4.62 billion, with a penetration rate of 48.6% [1]. The proliferation of social networking sites (SNS) in today’s information economy has created a global interest for scholars and practitioners in managing online communication for brands. The expansion of social media transformed how individuals communicate, as it evolved into the primary platform for online social interaction and information dissemination [2]. Social media platforms are reshaping how mankind consumes news content, since users may now not only learn about recent events but also share their own stories and advocate for various causes and issues [2].

A significant percentage of the US population continues to rely on social media platforms to get their news [3]. Around half of all adults in the United States (53%) say they read news via social media “frequently” or “sometimes”, using a variety of different SNS. For instance, among 11 social media sites surveyed as a frequent source of news, Facebook ranks first, with over a third (36%) of Americans routinely accessing it. YouTube is the second most popular platform, with 23% of US adults routinely using it for news. A total of 15% of US adults use Twitter as their primary news source. Other social media platforms are far less likely to be a credible source of information. Around one-in-ten Americans or less report getting news daily via Instagram (11%), Reddit (6%), Snapchat (4%), LinkedIn (4%), TikTok (3%), WhatsApp (3%), Tumblr (1%), and Twitch (1%) [3]. Therefore, social media sites are critical communication channels and businesses frequently use them as a marketing tool [4], to spur innovation [5], and to demonstrate their sustainability. Globally, brands are leveraging social media communication to increase sales income by reaching out to new customers and improving the experience of existing ones [6,7].

The quantity and quality of information available on social media have enhanced interaction between individuals and provided significant opportunities to communicate, but it has also represented a challenge to the environmentally friendly brands’ communication specialists [8,9]. Customers can use social media to keep informed, share stories, and engage with members of specific social groups (i.e., environmentally friendly brands’ fans), thereby shaping their friends’ and followers’ cognitions, attitudes, and actions toward brands [10,11,12]. In this dynamic communication process, in which senders transform themselves into receivers and receivers into senders, brands can lose control of their communication strategy [13,14,15,16] and the accuracy of information becomes critical. Additionally, a tremendous amount of information is spread on social media as news is dubious and, in some cases, intentionally misleading—this type of content is frequently referred to as *fake news* [17]. Purveyors of fake news can rapidly spread malicious content on social media and influence customers’ cognitions, attitudes, and behaviors [10].

Brands can become victims of the rapid spread of fake news on social media [18] as they can generate brand crisis [19], endure reputational damage, and suffer huge financial losses because of rapidly spreading false information [10]. Social media has the potential to strengthen or destroy a brand [20]; customers often discus on such platforms their negative experiences with brands [21,22,23]. We based our research on the narrow definition of fake news, which is considered to represent the “intentionally and verifiably wrong or false news produced for the purpose of earning money and/or promoting ideologies” (p. 213, [13]). Therefore, fake news represents “any information that contains an intentionally and verifiably false information and that can mislead readers” (p. 213, [13]).

Therefore, the issue of fake news is quite real, as corporations, governments, and even individuals may create and share information (or news) to a broad audience quickly via social media [24]. The propagation of fake news about any type of brand has a substantial economic, social, financial, political, and even environmental impact [25,26]. Given the scale of the problem and the damage that fake news has the potential to cause, it is important to examine and understand how fake news spreads on social media to develop effective reactive communication strategies [27]. Popular brands such as Pepsi Co, New Balance, and Starbucks are usually victims of fake news [10], but to the best of our knowledge, there are no studies investigating the effects of fake news on environmentally friendly brands. Therefore, we aimed to focus the investigation on such environmentally friendly brands which people buy or often prefer.

The literature lacks in explaining why people continue to share fake news on social media about brands. Although scholars have proposed different theories to explain various aspects of social media users’ behavior (i.e., selective exposure theory, uses and gratifications theory, social comparison theory, rational choice theory, or self-determination theory), these attempts only partially elucidated the spread of fake news on social media [10,28,29]. Despite the widespread dissemination of fake news and its negative consequences, the motivations for this detrimental behavior remain mostly unknown and under-researched [29]. There is a lack of theoretical frameworks on understanding fake-news-sharing behavior (knowingly or unknowingly) [28]. Psychological research is categorically required to better explain why people purposefully or inadvertently distribute fake news [30]. Given the importance of social media as a channel for communication, there is a gap in our understanding of fake news from the consumers’ perspective [31].

Social media users often experience a flow state while browsing SNS, and their experience influences the sharing behavior. The level of trust in the shared content might enhance or inhibit their fake-news-sharing behavior on social media. The current study aims to fill that gap by proposing flow theory [32,33] from positive psychology to be used as a theoretical framework to explain users’ behavior of sharing fake news about environmentally friendly brands on social media. Therefore, the aim of this paper is investigating why social media users share fake news online about environmentally friendly brands.

The novelty of this study lies in exploring the relationship between *users’ optimal experience on social media*, also called *social media flow* [34], and their sharing behavior of fake news about environmentally friendly brands. These relationships were not previously tested and, to our knowledge, no earlier study on fake news has used structural equation modeling (SEM) to explore the sharing of fake news using flow on an emerging market. The investigation was conducted in the Romanian emerging market, where the broadband internet speeds rank fourth worldwide [1], the internet penetration rate [35] and the number of social media users are high [1], and thus can be considered a quite relevant international benchmark for social media usage.

The paper is structured as follows: Section 1 presents the flow theory, while Section 2 contains the literature review, but also develops the research hypothesis and presents the conceptual model. Section 3 follows with the research methodology, while Section 4 reports the results of the investigation. Section 5 contains the discussion and a critical assessment of the results. The paper concludes with theoretical implications for the flow theory and fake news literature, managerial implications, as well as limitations and future research perspectives.

## 2. Flow Theory in the Social Media Context

The flow concept was first proposed by Csikszentmihalyi [32] after many years of research into play activities, creativity, and artists’ personality [36,37,38]. He [32] was interested to understand what makes daily human activities performed by different professionals (i.e., hockey and soccer players, spelunkers and explorers, mountain climbers, handball players, long-distance swimmers, surgeons, and chess players) inherently motivating and enjoyable. The participants in Csíkszentmihályi’s [32] interviews frequently described the experience as being carried along by the water current, an insight that determined him to call this state *flow*. Flow is defined as being a “holistic sensation that people feel when they act with total engagement” (p. 36, [32]). Flow can be considered an “optimal experience” [39] because “flow is defined as a psychological state in which the person feels simultaneously cognitively efficient, motivated, and happy” (p. 277, [40]).

In positive psychology, flow is not only a theory of intrinsic motivation [32], but also a theory of creativity [33,41,42], of holistic personal development [43], an important factor in the evolution of bio-culture and selection [44], and a theory for psychological rehabilitation practice [45,46]. In marketing communication studies, flow theory is considered a motivational paradigm [10].

Since flow varies in duration, frequency, and intensity [47], it is a continuous variable [48]. Flow can be experienced while engaging in a multitude of offline activities such as performing music or sports, reading a book, and shopping, or while engaging in online activities such as gaming, chatting with friends, browsing social networks sites, or purchasing. The most comprehensive description of the flow construct that distinguished it from the rest of everyday life activities is based on the following major characteristics [32,33]: distinct and clear goals, temporary loss of self-awareness, distorted sense of time, actions that merge with awareness and immediate feedback, intense concentration on the task at hand, a high level of control, a balance between the person’s available skills and the task challenges, and autotelic experiences.

Social media flow can be considered a multidimensional, aggregate construct, based on different concepts that together constitute the flow, such as: *time distortion* [10,49,50,51], *intrinsic interest* [32,52,53], *perceived control* [49,50,53], *concentration* [49,50,51,54], and *enjoyment* [32,51,54,55]. A bibliometric analysis of flow theory from 1975 to 2022 concluded that the five most used social media flow dimensions are [56]: *time distortion*, intrinsic interest, perceived control, concentration, and enjoyment.

*Intrinsic motivation* is specific to those individuals that are driven by satisfying internal rewards rather than relying on external pressures or extrinsic benefits [57]. Flow theory is based on intrinsic motivation [32], this dimension being essential for the flow experience. When people are in the flow state, they find the action intrinsically interesting as they engage in an activity for its own sake and enjoyment rather than for utilitarian purposes. A prerequisite of attaining flow is users’ motivation. Social media users browse because they enjoy the interaction with the computer, friends, and followers. In a flow state, engagement in a task is determined by intrinsic motivation. The task entails us simply because it is interesting, enjoyable, involves a level of challenge close to existing skills, and provides immediate feedback [32]. The satisfaction from performing that activity motivates the individual to continue.

*Perceived control* is defined as an individual’s belief that they are capable to influence and to make a difference in their life events [58] and obtain a specific desired outcome. Perceived control refers to “people’s perception of ease or difficulty in performing the behavior of interest” (p. 183, [59]). Flow occurs when a person perceives a high level of control while performing a task [32] (i.e., “people feel to be in control of their own actions, also being in the same time masters of their fate” (p. 3, [44]). In the online environment, perceived control relates to how web users perceive their ability to navigate successfully around the web environment and how the web responds to their inputs [60]. Technologies create this sense of control by responding to an individual’s feedback in ways that more static technology cannot [61]. Social media generates a sense of perceived control for users because it offers them responsive interactive elements, such as chat, comment, poke, like, and share [51], which they know how to use [62], and allows them to sign in and sign out whenever they choose.

*Concentration* is the degree to which one is focused on or paying attention to the task at hand [63,64]. Individuals achieving flow are extremely concentrated on the present moment [60]. This characteristic is significant for experiencing flow because “enjoyable activities require entire concentration on the task at hand—leaving no room in the mind for unnecessary information” (p. 58, [32]). In a flow state, a person selects a specific range of information that is allowed into awareness, and “that’s all that matters” (p. 58, [32]). Social media constitutes a perfect platform for individuals to concentrate on the task at hand due to the novelty and relevance of the information displayed following users’ preferences [34]. The screen reduces the range of stimuli to which the user is exposed, making it easier to focus because all the attention is concentrated on the relevant stimuli.

Csikszentmihalyi [33] considers *enjoyment* to be a common characteristic of flow experience. Individuals in flow “feel a sense of exhilaration, a deep sense of enjoyment” (p. 3, [44]) during an activity when they perceive a balance between their skills and the challenges of a situation. Perceived enjoyment can be defined as the degree to which users believe utilizing a virtual environment to be enjoyable regardless of performance repercussions [64]. Web enjoyment appears to be linked to the joy of discovery: finding, learning, or observing something for the first time [47].

In flow, individuals sense *distortion in time perception* [32] and they are unaware of time passing. A person experiencing flow is so absorbed in the activity at hand that they lose track of time [32]. Social media users spent an extensive amount of time during social media use because of the immersive and rewarding nature of online platforms [51]. The frequency of social media use is an important indicator of social media’s stickiness [51]. In social media, stickiness is important because it allows users to browse and view more content [65]. Given the rising amount of time spent on them, SNS are ideal for examining users’ flow experiences [66,67,68,69].

## 3. Hypothesis and Conceptual Model Development

### 3.1. Social Media Usage, Information Sharing, and Motives for Online Sharing News

Social media is conceptualized as a collection of web-based apps that are built on the conceptual and technological foundations of Web 2.0, enabling the creation and exchange of user-generated content [22,70]. Social media platforms include different social networking sites that allow people to interact with each other and share information about products and brands [71,72], such as networks (i.e., Facebook, Twitter, and Instagram), wikis (i.e., Wikipedia, Wikitravel, and Wiki-How), video sharing sites (i.e., YouTube, TikTok, and Vimeo), bookmarking sites (i.e., Twitter, Pinterest, and Reddit), virtual worlds (i.e., Second Life, Minecraft, and OurWorld), and rating sites (i.e., Google Customer Reviews, Yelp, and Foursquare). Social media usage can result in active and passive interaction [73]. Active social media usage refers to online activity that allows direct exchanges between users, such as commenting, sending messages, and interacting with other users in different ways [74]. Passive usage is defined as the monitoring of others without direct interaction [75].

Social media is a critical source of information since it enables users to get data fast and easily [23,51]. Due to the way information is displayed on social media in ‘thin slices’ [13], consumers have difficulty determining its authenticity and reliability [76]. Usually, headlines are designed with the express purpose of capturing people’s attention and are more important than the source of the information [77]. Because users may share and re-share content, genuine sources of information appear to be less reliable on social media [78,79]. This means that social media platforms are ideal to create, consume, and exchange all sorts of information, including fake news [80,81]. As a result of this lack of editorial monitoring [82], misleading claims can spread substantially wider, quicker, deeper, and more widely than true news [83]. Users frequently share, retweet, and distribute information on social media without fact-checking [84]. In this complicated informational landscape, social media usage increases the users’ exposure to inaccurate information about environmentally friendly brands. Therefore, the following hypothesis can be posited:

**Hypothesis** **1** **(H1).** *Social media usage has a positive influence on users’ exposure to inaccurate information on social media about environmentally friendly brands.*

Social media usage is one of the most popular online activities worldwide [85]. Although individuals initially engage in social media having an intrinsic or extrinsic motivation, during browsing, motivations can switch and/or expand. Often, users lose themselves while browsing content, chatting with friends, playing games, different new motives appear because of social media usage. The primary motivations for distributing (fake) news may be altruism [29,86], ignorance and entertainment [29], pass time, socialization, or information sharing [86]. The interactive nature of social platforms and the time spent creates opportunities for users to engage in new activities, including consuming and sharing news about environmentally friendly brands. Therefore, we hypothesize that:

**Hypothesis** **2** **(H2).** *Social media usage has a positive influence on users’ motives in sharing news about environmentally friendly brands.*

The information encountered on social media about environmentally friendly brands, also called green brands, could be problematic for users to distinguish accurate information from inaccurate one [87,88]. Without recognizing the spurious content, the exposure to new (misleading) information can motivate benign users to share it. In some cases, even the malicious users that identify the content as being fake can share it due to a vast range of possible motivations, such as to inform others, to know other users’ perspectives, to influence other users, to spark discussions, to entertain other social media users, to feel like they belong to a group, to demonstrate their knowledge about a specific topic, to please or to irritate others [22,89]. Therefore, in benign and malicious users’ cases, the exposure to inaccurate information creates the opportunity to determine new motivation in sharing news about environmentally friendly brands. As a result, it can be hypothesized that:

**Hypothesis** **3** **(H3).** *Exposure to inaccurate information on social media has a positive influence on users’ motives in sharing news about environmentally friendly brands.*

### 3.2. Online Trust

Lack of trust has been frequently regarded as one of the most severe impediments to people interacting with different websites [90], including social networking sites. Trust is a complex and abstract concept and defining it and operationalizing seems to be challenging [91,92]. Trust refers to the willingness to believe someone based upon positive expectations from past behavior [93,94]. It can be considered that online trust is an antecedent at the individual level that can influence users’ motives in sharing news about environmentally friendly brands. Different levels of online trust could influence the occurrence of different motivations among social media users in sharing news. An individual with a high level of online trust may be driven to disseminate information on social media to maintain authority among other users or to communicate with other followers of an environmentally friendly brand. Therefore, the following hypothesis can be postulated:

**Hypothesis** **4** **(H4).** *Online trust has a positive influence on users’ motives in sharing news about environmentally friendly brands.*

Only a few studies have examined the relationship between online trust and flow experience. Trust ameliorates users’ hesitancy to pay attention to the system, as the perceived risk of using the system would interfere without trust [95]. As a result, trust eliminates uncertainty or tension and facilities the flow experience. When the user’s mind is overwhelmed by uncertainty or danger, it is difficult to enjoy the flow because the flow is a state of complete involvement [32]. Social media users might feel completely involved when they have a lack of online trust because trust has long been viewed as means of coping with uncertainty [96]. Trust constitutes a substitute for control because individuals feel more secure and in control when interacting with someone they trust [96]. Therefore, online trust can facilitate perceived control, which is a key dimension of the social media flow experience. Based on the above analysis, the following hypothesis is proposed:

**Hypothesis** **5** **(H5).** *Online trust exerts a positive influence on the Social Media flow.*

Trust influences flow experience, while both factors predict the use intention and the actual online behavior. Trust minimizes ambiguity and consumers’ perceived risk, hence determining action [97]. Trust is created in an online environment because of successful information exchange [98] and represents an important factor in the information sharing process [99,100]. The issues of who and what to trust has grown in importance in the social media context, especially due to user-generated content and the speed of interaction [101]. Each day, social media users encounter dozens, if not hundreds, of pieces of user-generated information, and some of it may require trustworthiness evaluation. Trust information may assist a user in making important decisions, sorting, and filtering information, receiving suggestions, and developing a context within a community regarding whom and why to trust [101]. Individuals may feel comfortable sharing any news received from trusted sources, even if it seems to be fake [28]. The news fact-checking process could lack. In their excitement to continue sharing content, users may neglect the importance of checking every information received and may share it without verification in the name of speed and popularity [102].

Messages, including fake news, received from trustworthy sources are more likely to be propagated in social media and lead to increasing the organic reach [79]. Information given by a trusted source is more likely to be shared with other social media users even though is inaccurate [103]. Social media users share fake news without authentication due to online trust and that online behavior has tremendously negative effects on organizations and brands [28]. As a result, the next hypothesis can be proposed:

**Hypothesis** **6** **(H6).** *Online trust has a positive influence on sharing fake news about environmentally friendly brands on social media.*

### 3.3. Social Media Flow and Users’ Motives in Sharing News about Environmentally Friendly Brands

Social media plays an interactive and participatory nature that enhances the sense of immersion, perceived control, favors concentration, and offers rapid feedback to users becoming an ideal environment for experiencing flow [104,105,106] due to the great amount of time that individuals spend using it [66]. The flow experience that occurs during social media use is often referred to in the literature as *social media flow* [34]. Social media users engage in online browsing by having a clear and distinct goal in mind, such as to search for information about environmentally friendly brands and share it with their friends. An act has more than one motivation—therefore a drive, desire, wish, need, and goal are all defined as motivations [107]. From a broader perspective, when individuals initiate an activity, they may pursue an external goal (i.e., extrinsic motivated) or they may engage in actions for their reason, regardless of any external incentive system (i.e., intrinsic motivated) [22,108]. Individuals experiencing flow, are usually intrinsically motivated by the activity itself [32,66,68]. However, when they first engage in the task, they can be also extrinsic motivated [109,110] by rewards or to avoid punishments or penalties [111,112]. Intrinsic and extrinsic motivations are important antecedents of flow experience experienced while browsing mobile social network sites [68,110].

Motivation has also been considered as an antecedent of flow experience [68,109,110,113]. Motivation as an antecedent of situational involvement in a task constitutes a prerequisite for experiencing the flow in computer-mediated environments. Online consumer behavior is influenced by both goal-directed and non-directed motives [66], motivation being an essential factor in experiencing flow. Moreover, motivation was found to have a significant relation with users’ involvement on SNS pages (i.e., Facebook and Twitter) [114].

Users’ motives in sharing news indicates that benign social media users (i.e., those who are not motivated political, ideological, or by financial gain as malicious ones and are unable to recognize the veracity of the shared information) share information for three main reasons: (1) self-enhancement, to be recognized as an expert or knowledgeable by other users [115], (2) social motivation, to engage with their community and feel part of a group [116] and (3) altruistic motives, to show concern for others [117] and to try to help others [118]. It can be considered that users’ motives in sharing news about environmentally friendly brands can be a prerequisite for experiencing social media flow. Therefore, it can be hypothesized that:

**Hypothesis** **7** **(H7).** *The users’ motives in sharing news about environmentally friendly brands positively influence the social media flow.*

Flow theory has been considered a critical component for explaining online behavior [51,63,66] and determining the stimulating quality of online experiences, particularly for social media use [51]. Literature outlines that flow experience has been proven to influence online consumers’ attitudes, behavioral intentions, and behavior [119]. Social media constitutes a major source of information and assessing people’s flow experiences while browsing it may be more relevant and instructive than studying their experiences when using traditional websites [120].

### 3.4. Social Media and Fake News

Fake news is today considered to be one of humanity’s greatest threats, impacting many sectors of society, including the economy [121]. Although it has an impact on all aspects of life, it is particularly problematic in the environmental and health care sector, where it can delay or hinder adequate care, in some cases putting people’s lives at risk [122]. Fake news is not a new phenomenon [123,124], but in recent years, due to proliferation of the internet and social media, it has become more popular than ever [27]. From the telegraph in the 19th century to contemporary social media algorithms, new technologies have facilitated the spread of fake news [124]. Fake news is produced and spread on an exponential scale online, especially on social media. The growing concern among politicians and managers about the potential negative impact of fake news sparked the interest of scholars in studying it. A systematic literature review regarding fake news in social media and marketing identified a total number of 117 interdisciplinary studies [31].

Despite fake news is being commonly found on malicious websites, social media platforms such as Facebook, Twitter, WhatsApp, and Telegram are popular for the rapid transmission of dubious content [29]. In terms of popularity and engagement on social media, fake news exceeded actual news [125], and it became critical for brands to understand how this proliferation could hinder their marketing efforts [126]. There are seven types of fake news [127]: (1) *satire or parody* (i.e., when authors use irony and exaggeration to be comedic rather than to harm receivers), (2) *misleading content* (i.e., when authors use the information to present an issue or individual in a distorted perspective), (3) *imposter content* (i.e., when the message’s source pretends to be someone else), (4) *fabricated content* (i.e., when authors disseminate completely false information with the intent to deceive and cause damage), (5) *false connection* (i.e., when the headline, visual elements, and/or factual substance of a news item have no logical relationship), (6) *false context* (i.e., when authors blend true and inaccurate contextual information), and (7) *manipulated content* (i.e., when authors alter genuine information or imagery to deceive others). In many cases, malicious sources combine these types of content, resulting in more effective hybrid forms of fake news. The complex process of creating fake news, the extent to which fake news can be produced and replicated, the vast variety of fake news formats (i.e., texts, pictures, video clips, infograms, memes, gifs, etc.), the efficiency and speed through which fake news is propagated on social media, and the high level of commitment shown by those exposed to it (expressed through likes, shares, comments, recommendation or purchasing) have all turned fake news into a pervasive phenomenon [27].

In contrast to *news*, defined as “independent, reliable, accurate, and comprehensive information” about an organization (p. 11, [128]), *fake news* is inaccurate, false, or grossly distorted information presented as news to deceive the audience [129]. Fake news can also be conceptualized as “the presentation of false claims that purport to be about the world in a format and with a content that resembles the format and content of legitimate media organizations” (p. 20, [130]) or as the information that is designed to be confused with legitimate news and is intentionally false [131]. Fake news articles are intentionally fabricated to be deceptive and can be proven that they are false [132]. Fake news is also associated with the “intentionally and verifiably wrong or false news produced to earn money and/or promote ideologies” (p. 213, [13]), being able to “intentionally and verifiably false and could mislead readers” (p. 213, [13]).

To experience flow, social media users must be able first to concentrate on the task and perceive that they can complete it. Considering the peculiarities of social media sites (i.e., interactivity, instant feedback, and information richness), individuals must perceive a balance between available capabilities and task challenges and must receive immediate feedback [44,106,133,134,135,136]. In this process, social media users concentrate solely on the task at hand and act with deep, effortless, and total involvement [44,51,137]. Being an enjoyable experience [106,138], they perceive a sense of control over their activities [44,51,106], and become immersed in the activity due to self emerges with action [44,51,106,139,140,141]. A distortion in time perception and the duration of time is altered, hours pass in minutes, and minutes might expand to appear as hours [44,51,62,142]. As a result of the flow experience, social media users can engage in sharing information, including fake news about environmentally friendly brands. Previous research has broadly applied flow theory, particularly in the context of social media usage behavior [55,106]. Flow theory has been used to explain health-related social media sharing behavior [143], but no model has yet been empirically tested. Therefore, it is reasonable to adopt flow theory and examine its impact on social media users’ behavior of sharing fake news about environmentally friendly brands, so it can be inferred that:

**Hypothesis** **8** **(H8).** *Social media flow has a positive influence on sharing fake news about environmentally friendly brands on social media.*

## 4. Research Methodology

### 4.1. Research Design and Research Context

**Figure 1:**
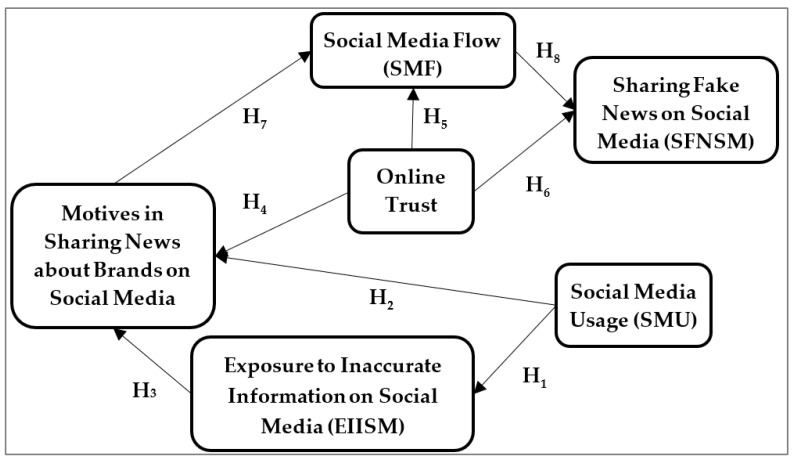
Theoretical model: Prerequisites of Sharing Fake News on Social Media.

The aim of this research was to determine the prerequisites that generate *sharing fake news online about* *environmentally friendly brands* by considering *social media flow* to be an important antecedent of users’ behavior. Specifically, based on the theoretical developments, the research model from Figure 1 is proposed to analyze the mediated impact of social media flow on sharing fake news about environmentally friendly brands on social media. *The social media flow* is generated by social media usage, exposure to inaccurate information on social media, motives in sharing news about environmentally friendly brands, and online trust. Figure 1 depicts the proposed theoretical model.

The research is based on an empirical investigation that relied on a quantitative survey via online interviews administered between November and December 2021 in Romania. The authors’ choice for conducting the investigation on the Romanian emerging market is justified as the country ranks 4th (232.17 Mbps) worldwide in the fastest Broadband Internet Speeds in 2021, after Monaco (261.82 Mbps), Singapore (255.83 Mbps) and Hong Kong (254.70 Mbps), before UK, France, Germany, or the USA [35]. The internet penetration rate in 2021 was estimated at 80%, while from about 19 million inhabitants 12 million were heavy social media users [1]. The yearly increase 2020–2021 of social media users is estimated at 1 million (+9%), while the number of “mobile connections” was in 2021 the equivalent to 135.6% of the total population [1], meaning that social media users are connected on about two devices on Facebook, Instagram, TikTok, etc. Therefore, Romania can be considered a quite relevant international benchmark for social media usage and for conducting this investigation, as it represents a valuable research context for both theory and practice.

The aim was to conduct a rigorous investigation on the internet and social media usage, according to quota samples from the Romanian national statistics based on age and gender [144]. The challenge was to find updated national statistics about the internet penetration and social media usage. The Romanian National Statistics [145] does not yet report the breakdown of internet and/or social media users on gender, age, etc., thus the authors could only rely on the estimations of international organizations [1]. As there is a lack of information regarding the exact number of Romanian males and females using the internet and social media, as well as their age, the authors opted for convenience sampling, thus aiming at coming close to the national breakdown on gender. Therefore, emails have been sent by the authors to social media users, via e-mail distribution groups of students, but also posted on social media platforms such as Facebook, Instagram, LinkedIn, etc. A total of 986 responses were collected and 922 responses were valid and retained after data quality checking (questionnaires with missing data were dismissed as in some cases respondents dropped filling in all measures). At the beginning of the survey, respondents were asked to think of any environmentally friendly brand they like most or they buy most frequently and to assess all items according to these brands.

Social media users (see Table 1) comprised 55.7% females (N = 524) and 44.25% males (N = 408). Most social media users were well educated: 0.1% were out-of-school (N = 1), 0.8 graduated the primary school (N = 7), 5.1% gymnasium (N = 47), 10 classes 7.6% (N = 70), vocational school 6.3% (N = 58), high school 40.6% (N = 374), college 5.6% (N = 52), university 22.8% (N = 210), postdoctoral studies 11.2% (N = 103). In total, 45.8% of respondents (N= 445) were less than 30 years old, 44.1% (N = 430) between 30~50 years, while 10.1% (N = 70) had more than 50 years. The mean age was of 32.36 years. In total, 43.5% of respondents had a low income, under the minim income in Romania (N = 401), 47.6% earned a middle income—between the minimum and the average income (N = 439) and 8.9% a high income—above the average income (N = 82).

### 4.2. Questionnaire Design and Measures

The questionnaire was developed starting with the relevant literature. The items of the questionnaire were taken from different scales, as recommended by the literature [146]. We used a five-point Likert scale (ranging from total disagreement to total agreement) and adapted the scales to the given research context, as being presented in Table 2. Six main constructs were delimited, namely Social Media Usage (SMU) (reflective construct comprising 3 items), Exposure to Inaccurate Information on Social Media (EIISM) (reflective construct comprising 1 item), Motives in Sharing News about Brands on Social Media (MSNSM) (reflective construct comprising 7 items), Online Trust (OT) (reflective construct comprising 2 items), Social Media Flow (SMF) (reflective construct comprising 5 items) and Sharing Fake News on Social Media (SFNSM) (reflective construct comprising 5 items).

## 5. Results

### 5.1. The Evaluation of the Measurement Models

The conceptual model and the developed hypothesis were analyzed relying on structural equations modeling in SmartPLS 3.0 (see Figure 1). All reflective constructs were checked for validity and internal consistency, the item loadings, average variance extracted (AVE), reliability indicators, and discriminant validity are computed in Table 3. All loadings are above the minimum thresholds of 0.70, suggesting that all measured items have convergence validity [149]. The minimum and maximum values range between 0.727–1.000, fulfilling the minimum thresholds. Reliability was tested using Cronbach’s α, which must exceed the threshold of 0.7 to be acceptable for confirmatory purposes [150]. All reliability values are above 0.7, confirming the internal consistency of the model. All AVE values are above 0.5, which indicates an adequate model [151] and support the convergent validity of the constructs. The composite reliability (CR) also suggests the reliability of the constructs, with the composite values being greater than 0.7 [149].

To test the discriminant validity of each construct the Fornell–Larcker and heterotrait–monotrait criteria were used (Table 3). Based on the Fornell–Larcker criterion [149,150], for each latent variable AVE value is higher than the correlation coefficient between the competent and all the distinct variables.

To avoid the possibility that the constructs are conceptually similar, the HTMT criteria was taken into consideration. The threshold value is 0.9 [152]—in the present study all constructs values are below 0.9, indicating the discriminant validity of the constructs (Table 4).

The level of collinearity of the items in the measurement model for the dataset was further addressed. The VIF value of all indicators is below 5, which is considered the threshold in the collinearity analyses [153]. The highest value is 3.411 (SFNSM3 item) for the dataset, indicating there is no multicollinearity. Next, a bootstrap procedure was applied to test the hypotheses and the relationships between the latent variables. All hypotheses were accepted with a significant, positive relationship based on t-statistics.

### 5.2. The Evaluation of the Structural Models

To perform a full analysis of the structural model, it was necessary to examine the constructs’ collinearity. The highest VIF value of the inner model is 1.063 (SMF→SFNSM), thus below the threshold value, indicated that there is not any multicollinearity between constructs. The goodness of fit of the saturated model is also acceptable. The square root mean residual (SRMR) has a value of SRMR = 0.051 which fulfills the recommended criteria < 0.08.

**Figure 2:**
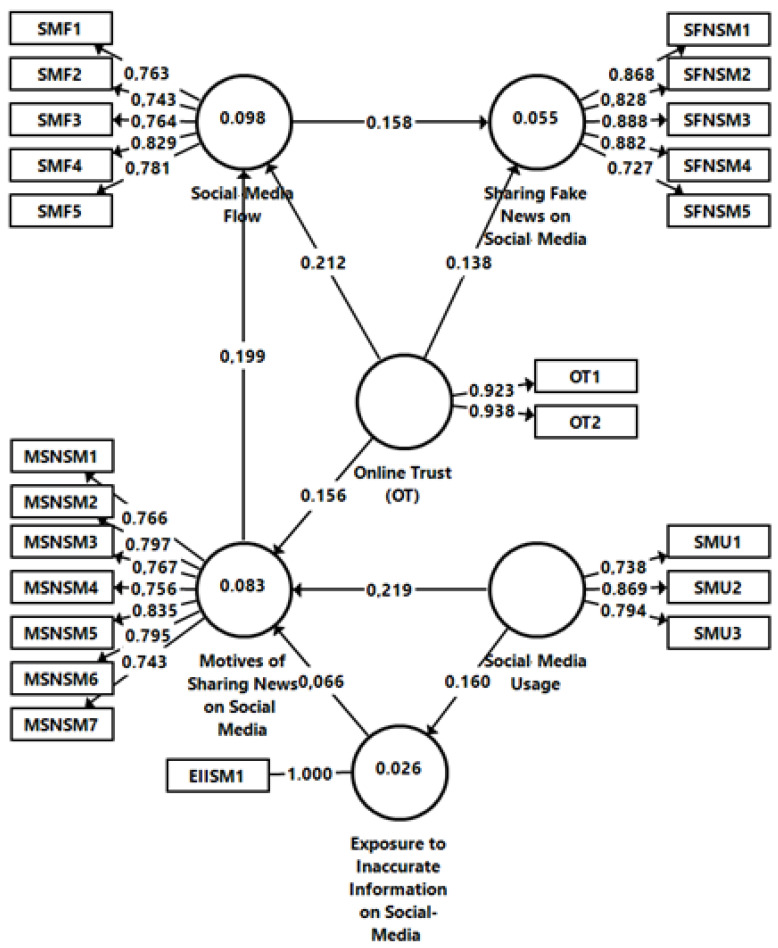
Structural model: Prerequisites of Sharing Fake News on Social Media.

Besides, *Social Media Usage* explains 2.6% of the variance of *Exposure to Inaccurate Information on Social Media* (R^2^ = 0.026), while *Social Media Usage*, *Online Trust* and *Exposure to Inaccurate Information on Social Media* explain 8.3% of the variance in *Motives in Sharing News about Brands on Social Media* (R^2^ = 0.083). Furthermore, 9.8% of the variance in *Social Media Flow* (R^2^ = 0.098) is explained by *Motives in Sharing News about Brands on Social Media* and *Online Trust*, while 5.5% in the variance of *Sharing Fake News on Social Media* (R^2^ = 0.055) is explained by *Online Trust* and *Social Media Flow*, defining a moderate predicting power of the structural model (see Figure 2).

Table 5 indicates a positive significant effect between the *Social Media Usage* and the *Exposure to Inaccurate Information on Social Media*, i.e., dynamic nature of the social networks and the amount of time spent browsing enable users to engage in new activities, such as consuming inaccurate information (β = 0.160; T-value = 5.091 and *p* < 0.001). Therefore, H1 is supported. H2 assumed that the *Social Media Usage* has a positive influence on the *Motives in Sharing News about Brands on Social Media*. The results (β = 0.219; T-value = 6.143 and *p* < 0.001) confirms that there is a meaningful relationship between the *Social Media Usage* and the *Motives in Sharing News about Brands on Social Media*, therefore H2 is supported. H3 presumed that the *Exposure to Inaccurate Information on Social Media* exerts a positive influence on the *Motives in Sharing News about Brands on Social Media*. The results (β = 0.066; T-value = 1.197 and *p* < 0.05) prove that the relation is positive and partially significant, allowing us to partially accept H3. H4 assumed that *Online Trust* has a positive influence on the *Motives of Sharing News about Environmentally Friendly Brands on Social Media*, meaning that online trust can determine social media users to engage in online activities being intrinsic or extrinsic motivated. The obtained results (β = 0.156; T-value = 4.932 and *p* < 0.001) show the positive and significant influence of the *Online Trust* on the *Motives in Sharing News about Environmentally Friendly Brands*, thus, H3 is supported by the empirical data.

H5 presumed that the *Online Trust* exerts a positive influence on the *Social Media Flow* meaning that users’ online trust positively influences the optimal experience in social media, also called social media flow. This means individuals may feel comfortable to enjoy the activity, because of reduced ambiguity and perceived risk, and experience flow. The results (β = 0.212; T-value = 6.247 and *p* < 0.011) confirm that there is indeed a positive and significant impact of the *Online Trust* on the *Social Media Flow*, hence H5 is accepted. H6 assumed that *Online Trust* has a positive influence on *Sharing Fake News about Environmentally Friendly Brands on Social Media*, i.e., people feel secure spreading any news they get from credible sources about environmentally friendly brands on social media, even if it appears to be inaccurate. The results (β = 0.138; T-value = 4.009 and *p* < 0.001) show that there is a positive and strong relation between these constructs, hence H6 is also accepted. H7 presumed that the *Motives in Sharing News about Environmentally Friendly Brands on Social Media* has a positive influence the *Social Media Flow*, i.e., users’ different motivations determine the occurrence of the optimal experience while browsing SNS. The results (β = 0.199; T-value = 6.632 and *p* < 0.001) prove the positive and significant relation, thus allowing to accept H7. H8 presumed that the *Social Media Flow* has a positive influence on *Sharing Fake News about Environmentally Friendly Brands on Social Media*, i.e., flow partially explains users’ behavior of sharing fake news about environmentally friendly brands on social media. The results (β = 0.158; T-value = 4.800 and *p* < 0.001) prove the positive and significant relation, thus allowing to accept H8 (see Table 5).

## 6. Discussions

The proposed model, in which sharing fake news is a consequence of social media flow, is supported by a substantial body of flow literature. According to previous research, flow has been used to explain a variety of specific user behaviors associated with communication and information use in an online context, including the use of information, communication, or information technology [138,154,155], online information searches [47,156,157], online communication [158], online user behavior [159], mobile internet usage and continuity [97,160], or use of social media [51]. Since spreading fake news is a distinct type of behavior related with communication and use of information in an online context, the findings of our study contribute to the current knowledge.

The results of the research highlight that social media usage (SMU) has a positive influence on users’ exposure to inaccurate information on SNS about environmentally friendly brands (H1). The finding corroborates other scholars’ assumptions that social media is a significant source of information [51] and that authentic sources of information are less trustworthy on social media [79], exposing social media users to inaccurate information, including fake news [80,81]. It has been shown that social media usage has a positive influence on users’ motives in sharing news about environmentally friendly brands (H_2_). These results are in line with prior findings [29,161] suggesting that individuals can share fake news having different motivation (intrinsic or extrinsic) when using social media platforms. Our study demonstrates that individuals’ motivations for sharing news about environmentally friendly brands are influenced by their use of social media.

The results reveal that online trust has a positive effect on the social media flow (H5). This finding is consistent with prior research [95,96] and emphasizes that online trust is a prerequisite of users’ optimal experience while browsing on social media. Online trust had a positive influence on sharing fake news on social media (H6) which is consistent with previous findings [162,163,164]. In a systematic literature review regarding sharing health information across online platforms, Le et al. [143] outline that in 19 articles trust was emphasized as a necessary condition that enables users to share HRI (health related information) on social media, being a key metric when considering whether to share information. Therefore, our study presents similar findings but in the context of sharing fake news about environmentally friendly brands on social media. Existing research [10] indicates that user motivation has a positive effect on the social media flow.

The insight regarding users’ motives in sharing news about environmentally friendly brands positively influence the social media flow (H7) is consistent with previous research in the flow literature, which has identified motivation as a predictor of flow experience [66,68,109,110,113]. In the current study, social media flow proved to have a positive influence on sharing fake news about environmentally friendly brands on social media (H_8_). Sharing fake news is a particular behavior that occurs in the context of social media usage. Previous studies from flow literature indicate that optimal experience influences users’ behavior while browsing online [55,106]. The research builds up on previous findings, but also highlights an important contribution in sharing fake news about environmentally friendly brands on social media context. Flow theory has been used to explain the health-related information sharing behavior on social media [143]. A recent study [164] indicates important differences in perception of fake news among the young and the middle-aged generations in terms of apprehension, interpretation, relation to fake news and media literacy, which might be a possible future research direction.

Fake news caused financial losses not only to commercial brands, such as PepsiCo, New Balance, and Starbucks [10] but also to environmentally friendly brands. For instance, Patagonia was a massive target of fake Facebook ads for its products between February and July 2020. In this period, Patagonia received over 1500 complaints of fraudulent Facebook ads for its products and submitted 236 notifications to Facebook regarding the problematic ads. The fake ads spread extensively on social media, creating significant financial damage to brands because the consumers may believe they are purchasing low-quality items if they are unaware the goods are counterfeit. As a result, Patagonia paused its ad spending on Facebook, in protest of the way the platform was handling hate speech and bogus content [165].

In 2021, Ricco Kimborough, a TikTok user, posted a bogus video online showing “his” Tesla Model S Plaid’s airbag unit falling in his hand. The video quickly went viral. Although he apologized for the fake viral video, the negative Tesla story generated massive clicks and propagated quickly on social media, and most of the users missed the revised information because it did not go viral like the initial fake post. Moreover, Jalopnik, the anti-Tesla auto blog, covered the fake viral TikTok video from a non-Tesla owner as if it were factual [166].

The current state of the art regarding fake news consequences indicates harmful effects of this phenomenon on society, businesses, and consumers’ level. Each level of study focuses on distinct links between the target and spreaders of fake news, as well as the numerous sources of legitimacy for fake news. Most current study focuses on the larger social level, mostly on the consequences of political fake news on voters and, in turn, on policymakers [31].

Fake news is being used to influence consumers’ perceptions regarding companies or products [167,168,169]. For example, as soon as the Pfizer-BioNTech vaccine was released [170], it became the top target of fake news, conspiracy theories, and disinformation efforts (i.e., mRNA vaccines alter human DNA), reaching millions of users on platforms such as Twitter, Reddit, and 4chan [170]. The dissemination of fake news on social media is a primary source of vaccine hesitancy, which is one of the major dangers to world health [171]. Therefore, when a company is a victim of fake news, it must carefully design a response strategy to minimize the negative impact [172].

Companies may sponsor and legitimize fake news while also being tainted by affiliation [18]. When confronted with fake news, users are more inclined to believe it if it is funded by a well-known brand [167]. Consequently, fake news has a negative influence on customer brand attitudes. When a company’s advertisement appears alongside fake news or on a fake news website, customers’ perceptions of source credibility affect brand trust and, as a result, brand attitudes [173]. Additionally, the associations with fake news led companies to significant reputational problems [174]. Advertising, specifically programmatic online advertising, facilitates the interaction between businesses and fake news [167]. The method of automating the distribution and placement of information to capture web traffic is known as programmatic advertising [175]. Because companies are increasingly choosing online advertising, fake news producers are driven to produce more fake content for increasing web traffic. As a result, programmatic advertising and fake news reinforce each other, worsening the impact of fake news on corporate branding and consumer brand perceptions [173,175,176]. Fake news can have damaging consequences that go beyond marketing [31]. For instance, individuals’ media trust and capacity to recognize real from fake news are affected as an effect of exposure to fake news in mainstream media [177]. This can generate confusion regarding prior knowledge, concerns about whether it is correct, and reliance on incorrect information [178]. As a result, individuals can underlie their next actions and decisions on this incorrect information. This can have detrimental consequences in politics [13], health-related concerns such as vaccination [179], finance, stock markets, and marketing [180].

Elon Musk’s case (i.e., the CEO of Tesla, SpaceX, and The Boring Company) illustrates how tweets and Facebook posts can harm sustainable brand stocks, generating investor lawsuits and governmental investigations. The proliferation of fake news on social media has accelerated during the COVID-19 pandemic [181,182,183,184], contributing to the outbreak’s propagation by overwhelming the government health releases online, which are quickly shared and spread to global audiences. The COVID-19 induced propagation of fake news on social media [185,186,187], with false content shared [188,189,190] that had strong effects among audiences worldwide. Social media platforms were first designed to facilitate relationships between friends, but, nowadays, they have evolved into critical pathways for the production and dissemination of information and news [80,81]. Fake news stories shared via social media get entwined with journalism and become a major source of information, as they are perceived as genuine news by the public [191]. In this complicated context and considering the real consequences for companies, explaining users’ behavior of sharing fake news about environmentally friendly brands on social media becomes extremely important [29].

For instance, pharmaceutical companies such as Pfizer and Moderna have suffered significant damage because of rumors, conspiracy theories, misinformation, misleading connections, or manipulated content propagated through social media platforms [192]. During COVID-19, 46% of the United Kingdom’s population was exposed to COVID-19-related fake news [193], and more than 25% of the most popular COVID-19-related YouTube videos contained inaccurate or misleading content, reaching over 62 million views globally [194]. Apuke and Omar [86] confirmed the importance of online information trust in predicting fake news sharing related to the COVID-19 pandemic among social media users.

## 7. Conclusions

### 7.1. Theoretical Contributions

Previous research in the field of fake news used different theories to explain various aspects of social media users’ behavior (i.e., selective exposure theory, uses and gratifications theory, social comparison theory, rational choice theory, or self-determination theory), but in this paper, theoretical knowledge is extended based on the social media flow, as a basic construct deduced from flow theory. This represents a significant factor influencing fake-news-sharing behavior and implies that brand communicators should prioritize monitoring users’ experiences on social media to establish preventative and reactive communication strategies in response to the proliferation of fake news online. The research extends knowledge about the marketing outcomes of social media flow and contributes to a better understanding of the antecedents of sharing fake news about environmentally friendly brands on social media.

Furthermore, the conceptual model also investigates for the first time the relation between the exposure to inaccurate information on social media and users’ motives in sharing news about environmentally friendly brands (H3), and the influence of online trust on users’ motives in sharing news about environmentally friendly brands (H4). No other studies in the literature have been found investigating these effects which have been proven to be strong and significant, thus contributing to enhancing users’ sharing of fake news on social media. These findings represent a powerful and important original contribution of the paper.

Another important theoretical contribution of the paper lies in the fact that previous models have not linked the flow theory to spreading fake news via social media. This finding, investigated on an emerging market with a high number of new social media users, also represents a strong managerial contribution, as marketers from organizations need to be very careful about the news that their followers spread via social media, thus making it possible for inaccurate and/or false information to gain the rapid attention and trust of consumers who might not have the time and/or interest to check their reliability, thus causing potential harms to brands or products.

The current study contributes to the flow and fake news literature by providing new insights on the effects of social media flow and the factors that contribute to the spreading of fake news. The approach is unique in that it examines social media users’ optimal experiences and the effect on user behavior, such as sharing fake news. The suggested model (see Figure 1) is more parsimonious than existing models from the fake news literature since the flow experience is one in which social media users are cognitively efficient, motivated, and happy. Additionally, the proposed model explains social media users’ behavior in sharing fake news by considering new factors not previously examined in the literature.

Various psychological variables have been examined in the fake news literature, including individual motivation (i.e., sharing motives), attention, belief (i.e., influenced by delusionality, dogmatism, religious fundamentalism, bullshit receptivity, and overclaiming), reasoning style (e.g., analytic thinking, heuristics, or mental shortcuts), truth discernment, overconfidence, prior knowledge, the illusory truth effect, perceived source credibility, belief in news content, emotional reaction (positive or negative) to fake news, etc., to explain why social media users share fake news [195], but not the holistic experience (i.e., social media flow) that can occur during browsing SNS. Therefore, this study contributes to the psychology of fake news by emphasizing not only the cognitive or affective variables that influence this type of behavior, but also the holistic experience users have on social media, which requires the individual to be cognitively efficient, motivated, and happy at the same time.

Fake news spreaders can be non-human (i.e., social bots and cyborgs) or real human beings (e.g., social media users) [17] but it is more difficult to counteract human creators and spreaders of fake news than non-human representatives [10]. Therefore, it is crucial to better understand the psychological factors that predict the sharing behavior of fake news on social media. Therefore, we consider that the current model based on flow theory helps us to better understand the fake news activity on SNS.

### 7.2. Managerial Implications

Among the managerial contributions of the paper, it can be highlighted that brand managers are provided with a deeper insight into social media users’ behavior when it comes to distributing bogus news about environmentally friendly brands. Brand managers may develop a better understanding of how this type of deceptive content spreads on social media, so they can assess potentially risky information that could affect brands in real-time. Online-shared fake news has the potential to harm brands; thus, practitioners should focus on leveraging social media platforms to counteract fake news, such as by converting SNS users into endorsers or spreaders of genuine information. Additionally, marketers should motivate social media users to detect potential fake news disseminated about brands and act as gatekeepers.

Brand managers should also educate customers to evaluate the accuracy of brand information shared via social media. This means that promoting media literacy could be a critical proactive strategy for reducing the unintended social media spread of bogus news. Enhancing social media users’ awareness of fake news and its detrimental consequences on brands may result in a decrease in sharing activity. The brand manager should persuade clients to verify information using brand websites or legitimate sources. In this regard, AI technology can be used to immediately detect and inform communication specialists, thereby limiting the propagation of unverified news to social media users.

### 7.3. Limitations and Future Work

The research has several limitations that should be considered in the context of future perspectives. Because the study was conducted in a single country, the findings cannot be generalized to other social contexts, and the results must be interpreted in the given cultural setting. Future research should focus on testing the proposed model in other geographical and/or cultural regions throughout the world. The conceptual model has been tested for environmentally friendly brands. Future studies could replicate the model by considering environmentally versus non-environmentally friendly brands, but also could rely on analyzing such brands in different industries (food versus non-food or fashion versus electronic articles, etc.). An interesting future research direction could also lead on determining the fake news spread intention of younger versus older people or of baby boomers versus generation Xers or millennials.

Another limitation of the research regards the fact that the items were measured using data from a self-administered questionnaire, which is a retrospective measure. Future studies could rely, for instance, on objective measurement methods for capturing cognitive and affective states, such as the experience sampling method (ESM), in which respondents are asked to describe their flow experience at random times during the day. As the research relied on convenience sampling, there are some more limitations characteristic of non-probability sampling. In this regard, the sample might not be representative for the basic social media user population. Future research could test the model using, for instance, probability sampling. Furthermore, the research model could be expanded in future studies by also including additional constructs such as social media users’ personality traits, sociodemographic characteristics (age and education), contextual variables (internet use and visit goals), the device(s) used to browse social media (smartphone, tablet, watch, or glasses), other users’ motivations for sharing fake news about brands, and platform characteristics (i.e., number of shares/retweets, number of likes, etc.), to better understand the fake-news-sharing behavior.

As the current research did not make any distinctions between intentionally sharing (with or without harmful intent) and unintentionally sharing fake news about environmentally friendly brands, future studies could focus on examining in detail these two contexts and shed light on social media users’ motivations of deliberately sharing bogus information vs. unknowingly sharing incorrect information about environmentally friendly brands. Future research could also consider fake news spread behavior on emerging markets versus developed ones, or on markets with high access to internet and increased internet speed versus markets with limited access to internet.

## Figures and Tables

**Table 1:** Socio-demographic characteristics of the sample versus Romanian statistics.

Dimension	Variable	Frequency	Percentage	Percentage	Deviation of Sample vs. Statistics *
				National Statistics *	
Gender	Female	524	55.75%	51.1%	+4.65
	Male	408	44.25%	48.8%	−4.55
Education	Out-of-school	1	0.1%	n/c	n/c
	Primary school	7	0.8%	n/c	n/c
	Gymnasium	47	5.1%	n/c	n/c
	10 classes	70	7.6%	n/c	n/c
	Vocational school	58	6.3%	n/c	n/c
	High school	374	40.6%	n/c	n/c
	College	52	5.6%	n/c	n/c
	University	210	22.8%	n/c	n/c
	Postdoctoral studies	103	11.2%	n/c	n/c
Age	<30 years	445	45.8%	31%	+14.8%
	30–50 years	430	44.1%	30%	+14.1%
	>50 years	70	10.1%	39%	−28.9%
Income	Low	401	43.5%	n/c	n/c
	Middle	439	47.6%	n/c	n/c
	High	82	8.9%	n/c	n/c

Note: n/c: not considered; * The Romanian National Statistics [145] only refers to the population breakdown according to gender and age-groups, and not to the internet or social media usage. Therefore, a quota sampling on these characteristics is not yet possible.

**Table 2:** Constructs and items.

	Item	Measure	Loading	Cronbach’s Alpha/AVE/CR	Source
Social Media Flow (SMF)	SMF1	While using social media, I am deeply engrossed.	0.763	0.836/0.603/0.884	Adapted from Kwak et al. [68]; Brailovskaia et al. [147]
	SMF2	While using social media, I am immersed in the task I am performing.	0.743		
	SMF3	Time flies when I am using social media.	0.764		
	SMF4	While using social media, I often lose track of time.	0.829		
	SMF5	While using social media, I often spend more time than I had intended.	0.781		
Sharing Fake News on Social Media (SFNSM)	SFNSM1	The news I shared on social media about environmentally friendly brands seemed accurate at the time, but later I found out it was made up.	0.868	0.895/0.707/0.923	Adapted from Chadwick and Vaccari [89]
	SFNSM2	The news I shared on social media about environmentally friendly brands was exaggerated, but I was not aware of this at the time of sharing.	0.828		
	SFNSM3	The news I shared on social media about environmentally friendly brands seemed to be real news at the time of sharing, but later I found out that was fake news.	0.888		
	SFNSM4	The news I shared on social media about environmentally friendly brands initially seemed accurate but was later proven to be a hoax.	0.882		
	SFNSM5	The satirical news I shared on social media about environmentally friendly brands was presented as real news.	0.727		
Online Trust (OT)	OT1	I trust the information that is shared on social media (Facebook, Instagram, Twitter, TikTok, etc.).	0.923	0.846/0.866/0.928	Adapted from Fang et al. [148]
	OT2	I trust the news that is shared on social media (Facebook, Instagram, Twitter, TikTok, etc.).	0.938		
Motives in Sharing News about Brands on Social Media (MSNSM)	MSNSM1	When I share news about brands on social media (Facebook, Instagram, Twitter, TikTok, etc.) is important to find out other people’s opinions.	0.766	0.893/0.609/0.916	Adapted from Chadwick and Vaccari [89]
	MSNSM2	… to influence others.	0.797		
	MSNSM3	… to provoke discussions.	0.767		
	MSNSM4	… to entertain others.	0.756		
	MSNSM5	… to feel like I belong to a group.	0.835		
	MSNSM6	… to demonstrate my knowledge.	0.795		
	MSNSM7	… to please others.	0.743		
Exposure to Inaccurate Information on Social Media (EIISM)	EIISM1	Over the last month, I come across news on social media that I thought was not fully accurate/authentic.	1.000	1.000/1.000/1.000	Adapted from Chadwick and Vaccari [89]
Social Media Usage (SMU)	SMU1	On average, I spend a lot of time browsing on Facebook.	0.738	0.721/0.643/0.843	Adapted from Chadwick and Vaccari [89]
	SMU2	On average, I spend a lot of time browsing on Instagram.	0.869		
	SMU3	On average, I spend a lot of time browsing on TikTok.	0.794		

Note: Factor loading > 0.7; Cronbach’s alpha > 0.7; average variance extracted (AVE) > 0.5; composite reliability > 0.7.

**Table 3:** Discriminant validity analyses (Forner-Larcker).

Construct	OT	EIISM	MSNSM	SFNSM	SMF	SMU
OT	0.931					
EIISM	−0.048	1.000				
MSNSM	0.161	0.094	0.781			
SFNSM	0.176	0.043	0.263	0.841		
SMF	0.244	0.107	0.233	0.192	0.777	
SMU	0.042	0.160	0.236	0.090	0.296	0.802

Note: OT: Online Trust; EIISM: Exposure to Inaccurate Information on Social Media; MSNSM: Motives in Sharing News about Brands on Social Media; SFNSM: Sharing Fake News on Social Media; SMF: Social Media Flow; SMU: Social Media Usage.

**Table 4:** Discriminant validity analyses (heterotrait–monotrait).

Construct	OT	EIISM	MSNSM	SFNSM	SMF	SMU
OT						
EIISM	0.053					
MSNSM	0.186	0.098				
SFNSM	0.201	0.045	0.295			
SMF	0.283	0.121	0.263	0.222		
SMU	0.069	0.189	0.291	0.110	0.378	

Note: OT: Online Trust; EIISM: Exposure to Inaccurate Information on Social Media; MSNSM: Motives in Sharing News about Brands on Social Media; SFNSM: Sharing Fake News on Social Media; SMF: Social Media Flow; SMU: Social Media Usage.

**Table 5:** The path coefficients of the structural equation model.

Paths	Path Coefficients	Standard Deviation	T-Value	CI ^1^	*p*-Value	Hypotheses
SMU→EIISM	0.160	0.032	5.091	0.100–0.277	0.000 **	H1-Supported
SMU→MSNSM	0.219	0.036	6.143	0.147–0.285	0.000 **	H2-Supported
EIISM→MSNSM	0.066	0.034	1.967	−0.006–0.130	0.050 *	H3-Supported
OT→MSNSM	0.156	0.032	4.932	0.096–0.218	0.000 **	H4-Supported
OT→SMF	0.212	0.034	6.247	0.147–0.278	0.000 **	H5-Supported
OT→SFNSM	0.138	0.034	4.009	0.076–0.205	0.000 **	H6- Supported
MSNSM→SMF	0.199	0.030	6.632	0.142–0.254	0.000 **	H7- Supported
SMF→SFNSM	0.158	0.033	4.800	0.094–0.221	0.000 **	H8-Supported

Note: * *p* < 0.05; ** *p* < 0.001; OT: *Online Trust*; EIISM: Exposure to Inaccurate Information on Social Media; MSNSM: Motives in Sharing News about Brands on Social Media; SFNSM: Sharing Fake News on Social Media; SMF: Social Media Flow; SMU: Social Media Usage. ^1^ CI = Confidence Interval (2.5–97.5%).

## Data Availability

Not applicable.
